# Underrecognition of Autism and Implications for Adult Psychiatric Practice: A Narrative Review

**DOI:** 10.7759/cureus.107164

**Published:** 2026-04-16

**Authors:** Alfredo de Jesús Garza Guerra, Elisa del Rocio Mata Cortes, Gabriela Hilian Adame Rocha, Ana Cecilia Montemayor Mancias, Stefan Mauricio Fernández Zambrano

**Affiliations:** 1 Psychiatry, Hospital Universitario Dr. José Eleuterio Gonzalez, Monterrey, MEX; 2 Psychiatry, Jalisco Institute of Mental Health and Addictions, University of Guadalajara, Guadalajara, MEX

**Keywords:** adaptation, adult, autism spectrum disorder, comorbidity, diagnosis, differential, mental health, neurodevelopmental disorders, psychiatry, psychological

## Abstract

Autism in adulthood remains underrecognized across psychiatric settings despite epidemiological evidence supporting its persistence across the lifespan. Recognition is often hindered by heterogeneous presentation, misattribution of autistic traits to co-occurring psychiatric conditions, camouflaging processes, and limited integration of developmental history in adult assessments. This narrative review aims to synthesize current evidence on adult presentation and co-occurring conditions, examine barriers to identification, and propose a developmental, dimensional, and contextual framework for psychiatric assessment and formulation. A narrative review guided by the Scale for the Assessment of Narrative Review Articles was conducted. Peer-reviewed literature published between January 2010 and December 2025 was identified through structured searches of PubMed, Scopus, and Web of Science, with additional sources identified through reference list screening. Studies were selected based on conceptual relevance and applicability to adult psychiatric contexts. The literature indicates that autism in adulthood is characterized by marked heterogeneity, with presentations often shaped by internalized features, compensatory processes, and co-occurring psychiatric conditions. Across studies, these factors contribute to diagnostic overshadowing, delayed identification, and fragmented care trajectories. Developmental, neurobiological, and service-level evidence further highlights the influence of contextual and structural factors on recognition within adult psychiatric settings. Autism in adulthood is best conceptualized within a developmental, dimensional, and contextual framework. Improving identification in psychiatric practice requires systematic attention to developmental history, internal experience, compensatory processes, co-occurring conditions, and structural barriers. Integrating these elements may enhance clinical formulation, reduce diagnostic fragmentation, and improve care for autistic adults within psychiatric services.

## Introduction and background

Historically, autism has been predominantly identified and researched within early childhood contexts, shaping recognition frameworks, clinical practices, and service structures oriented toward pediatric populations [1-3]. However, population-based studies indicate that autistic traits are expressed across the lifespan, and prevalence estimates in adults remain relatively stable [4-6]. Despite this continuity, recognition within adult psychiatric services remains inconsistent, contributing to delayed identification and fragmented clinical trajectories [1,7-9]. At present, the factors contributing to this underrecognition in adult psychiatric contexts remain conceptually fragmented across epidemiological, developmental, and clinical literatures. Incorporating autism more explicitly within adult clinical reasoning may support developmentally informed and longitudinally coherent psychiatric formulation.

Autism is a neurodevelopmental condition characterized by persistent differences in social communication and interaction across multiple contexts, together with restricted, repetitive patterns of behavior, interests, or activities, typically present from early developmental stages, although they may become more apparent later in life. In this article, autism is conceptualized as a multidimensional neurodevelopmental profile involving variation across social cognition, communication style, sensory processing, executive functioning, and regulatory patterns [2,10,11]. This formulation aligns with dimensional psychiatric models that situate neurodevelopmental traits along continua within the general population [2,10,11], as well as with contemporary neurodiversity-informed scholarship emphasizing heterogeneity and nonpathologizing frameworks [12,13]. This conceptualization extends beyond strictly deficit-based or categorical interpretations, while retaining clinical attention to functional impact, support needs, and contextual demands.

Together, developmental, dimensional, and contextual perspectives provide the conceptual foundation for the present review. A developmental lens situates autistic characteristics as enduring yet dynamically expressed patterns shaped by shifting environmental expectations across the lifespan [3,14,15]. From a dimensional perspective, autism may be understood as a distribution of neurocognitive traits present within the general population that acquire clinical salience when associated with significant person-environment mismatch, cumulative stress exposure, or barriers to participation [2,10,16,17]. A contextual perspective highlights how sociocultural norms, gendered expectations, and health system structures influence recognition, diagnostic pathways, and access to care [13,18].

Clinical relevance emerges not from neurodevelopmental difference per se, but from the dynamic interaction between an individual’s profile and environmental expectations, social norms, and structural barriers to participation [13,17,18].

Marked heterogeneity in trait expression, developmental trajectory, and adaptive profile contributes to more limited recognition in adulthood, particularly among individuals whose presentations diverge from recognition models historically derived from early childhood prototypes [1-3]. This includes many women, individuals without learning disability or language delay, and individuals who employ sustained compensatory or camouflaging strategies [19-22]. In such cases, observable behavioral indicators may remain subtle, while internal cognitive load and cumulative stress remain considerable [23,24].

Individuals recognized later in life refer to those whose autistic neurodevelopmental profile was not identified during childhood, often due to limited access to assessment, gender-related recognition biases, or the presence of co-occurring psychiatric conditions [1,8,9,25]. Many of these individuals develop compensatory strategies, understood as cognitive, behavioral, or regulatory mechanisms through which autistic individuals adapt to environmental demands despite underlying differences in social cognition, executive functioning, or sensory processing. These strategies may include explicitly learning social conventions, using analytical reasoning to interpret interpersonal interactions, developing structured routines to manage environmental demands, or relying on cognitive supports to regulate attention and sensory load [22,26-28]. In social contexts, such compensatory processes may contribute to social camouflaging, a broader construct referring to impression management strategies that can reduce the observable visibility of autistic traits [21,23,29].

Although awareness of autism across the lifespan has increased, adult psychiatric practice continues to face challenges in identification and formulation [1,7,9]. Autistic adults frequently present with co-occurring psychiatric conditions, without underlying neurodevelopmental differences having been previously integrated into clinical reasoning [7,30,31]. Diagnostic ambiguity may arise when symptom clusters are interpreted primarily from a cross-sectional perspective, without sufficient incorporation of developmental history [32]. As a result, some adults receive multiple psychiatric diagnoses before autism is considered within formulation, contributing to fragmented care trajectories and delayed access to appropriate support [7-9].

Terminology remains an important consideration in autism research and clinical discourse. In this article, the term “autism” is used in alignment with identity-first language preferred by many autistic individuals and advocacy groups, while recognizing diversity of linguistic preference within the community [33].

This narrative review examines factors contributing to underrecognition of autism in adulthood within psychiatric practice, integrating epidemiological, neurodevelopmental, clinical, and service-level perspectives. Specifically, it aims to 1) synthesize evidence regarding adult presentation and co-occurring conditions, 2) analyze conceptual, structural, and contextual barriers to recognition in adult psychiatric settings, and 3) advance a developmental, dimensional, and contextual framework to inform more coherent clinical formulation and service provision for autistic adults.

## Review

Methodological approach

This study was conducted as a narrative review guided by the recommendations of the Scale for the Assessment of Narrative Review Articles, which informed the clarity of aims, transparency of the search strategy, balanced interpretation of evidence, and clinical relevance.

A narrative review methodology was selected, given the heterogeneity of adult autism research, which spans epidemiological, neurobiological, clinical, and service-level domains. Accordingly, the objective was to provide a conceptually integrated and clinically relevant synthesis rather than quantitative aggregation or exhaustive systematic retrieval.

Search Strategy

A targeted literature search was conducted in PubMed, Scopus, and Web of Science to identify peer-reviewed studies on autism in adulthood and mental health. The search covered publications from January 2010 to December 2025, reflecting contemporary developments in adult autism research.

Search terms included combinations of keywords related to autism spectrum disorder, adulthood, mental health, psychiatric practice, co-occurring conditions, developmental trajectory, and service organization, as well as conceptually relevant terms such as camouflaging and underrecognition. Reference lists of relevant articles were also screened to identify additional sources. Studies published in English were included, consistent with the databases and search strategy employed.

Inclusion and Exclusion Criteria

Articles were included if they examined clinical, psychiatric, neurobiological, epidemiological, or service-level aspects of autism in adult populations (≥18 years) or included clearly defined adult subgroups. Studies were selected based on their relevance to adult presentation, recognition, diagnostic formulation, co-occurring conditions, or service organization.

Articles were excluded if they focused exclusively on pediatric populations without clear relevance to adulthood or lacked applicability to adult psychiatric practice.

Screening and Synthesis

Screening was conducted through title and abstract review, followed by full-text assessment of relevant studies. Study selection was performed by members of the author team, with discrepancies resolved by consensus.

Although a structured and transparent search strategy was implemented, a formal PRISMA flow diagram was not applied, as this review follows a narrative design prioritizing conceptual integration and clinical relevance rather than exhaustive systematic inclusion.

A total of 92 peer-reviewed publications were included and synthesized using an iterative thematic approach focused on adult presentation, developmental trajectory, co-occurring conditions, and service-level factors relevant to psychiatric practice.

Ethical Considerations

This study was based exclusively on previously published, publicly available, peer-reviewed literature. No primary data were collected, and no human participants, identifiable personal data, or confidential information were involved. Accordingly, formal ethical approval and informed consent were not required.

The review was conducted in accordance with principles of ethical scholarly practice, including accurate representation of sources, appropriate citation, and transparency in reporting. All authors adhered to standards of academic integrity in the preparation of this article.

Findings: thematic narrative synthesis

The following sections present the thematic narrative synthesis of the reviewed literature, organized into major domains relevant to the underrecognition of autism in adult psychiatric practice. These domains include epidemiological patterns, developmental and neurobiological influences, adult presentation, and co-occurring psychiatric and medical conditions.

Epidemiology

Autism represents one of the most prevalent neurodevelopmental profiles worldwide. Recent global estimates place prevalence between approximately one in 31 and one in 54 individuals (≈1.9%-3.2%), depending on surveillance methods and identification criteria [6,34]. In the United States, population-based studies have documented a marked increase in identified cases over the past decade [35]. This trend is generally understood as reflecting improved recognition and broader conceptualization rather than a true increase in underlying prevalence [6,35].

In adults, prevalence estimates are relatively consistent, typically ranging between 1% and 2% [4,5]. Broader global estimates across age groups range from approximately 1.9% to 3.2%, depending on surveillance methods and identification criteria [6,34]. Such variation likely reflects differences in methodology, case ascertainment, and population sampling rather than true differences in underlying prevalence. These findings support the lifespan expression of autistic traits and highlight limitations of historically pediatric-centered service models [2,3]. A substantial proportion of autistic adults remain unrecognized [1], particularly those with more subtle or camouflaged presentations, suggesting that current estimates may underestimate true population representation.

Epidemiological studies have historically reported higher identification rates in male patients. However, emerging evidence indicates that observed differences are influenced by detection bias, as well as differences in phenotypic expression and camouflaging strategies [19,21,22]. Recent meta-analyses suggest that the male-to-female ratio may be closer to 3:1 rather than earlier estimates of 4-5:1 [6,20].

There is also increasing recognition of greater representation of transgender and gender-diverse individuals within autistic populations [36]. However, the limited inclusion of gender identity variables in epidemiological studies restricts the precision of current estimates [13,36], underscoring the need for more inclusive approaches in adult psychiatric assessment.

Prevalence estimates vary across regions and are influenced by socioeconomic context, healthcare access, and diagnostic infrastructure [4,6,34]. Higher estimates are typically reported in high-income countries with established surveillance systems, suggesting that variability reflects differences in recognition rather than true prevalence. Lower estimates in other settings likely reflect underrecognition [4,6,34].

Important gaps also persist in racially minoritized populations, where underrepresentation may reflect structural barriers, disparities in access to care, and culturally mediated recognition processes [18].

Overall, epidemiological evidence supports autism as a heterogeneous neurodevelopmental profile expressed across the lifespan [2,3,6]. The discrepancy between estimated prevalence and formally recognized adult cases highlights a significant gap in recognition, with implications for psychiatric formulation, clinician training, and service development [1]. Emerging research further documents delayed identification, diverse recognition pathways, and limited access to postrecognition support in adult populations [8,9,25]. Table 1 summarizes key epidemiological domains shaping adult autism recognition and their implications for psychiatric practice within a developmental, dimensional, and contextual framework.

**Table 1:** Key epidemiological domains relevant to adult autism recognition and implications for psychiatric practice Prevalence estimates vary across study design, surveillance systems, identification frameworks, healthcare access, and sociocultural factors shaping recognition of neurodevelopmental diversity. Adult prevalence figures should therefore be interpreted cautiously and may underestimate true population representation, particularly in settings lacking structured adult assessment pathways

Domain	Epidemiological finding	Implications for adult psychiatric practice
Global adult prevalence	Population-based studies estimate adult autism prevalence between approximately 1%-2%, supporting the lifespan expression of autism [5,6,34]	Autism should be routinely considered in adult psychiatric assessment and developmental formulation
Increasing recognition rates	Identification rates have increased over the past decade, likely reflecting improved detection and expanded recognition criteria rather than true prevalence change [1,35]	Increasing adult identification highlights the need for responsive, lifespan-informed mental health services
Limited recognition in adulthood	A substantial proportion of autistic adults remain without formal recognition, particularly those with internalized or camouflaged presentations [1,8,25]	Delayed recognition may contribute to fragmented clinical trajectories, diagnostic overshadowing, and cumulative adaptive burden
Sex and gender differences	The male-to-female ratio may approximate 3:1, suggesting potential underidentification of women. Emerging research also indicates increased representation of transgender and gender-diverse identities among autistic individuals [23,36,37]	Clinicians should consider sex- and gender-related variation in presentation, including camouflaging strategies, minority stress exposure, and intersections with gender-affirming care pathways
Racial and sociocultural representation	Epidemiological data in racially minoritized populations remain limited. Underrepresentation may reflect structural barriers, disparities in healthcare access, and culturally mediated recognition processes [18,38]	Culturally responsive assessment frameworks and awareness of structural inequities may help reduce disparities in adult identification and access to care
Sociocultural and health system variation	Prevalence varies according to surveillance infrastructure, healthcare access, and cultural recognition of neurodevelopmental diversity [5,6,34]	Differences in service organization influence recognition patterns and reflect structural determinants of recognition and access to evaluation. Lifespan-oriented recognition approaches emphasize autism as a lifelong neurodevelopmental profile whose expression varies across developmental stages and social contexts
Recognition pathways in adulthood	Adults often experience heterogeneous routes to evaluation, including prior psychiatric diagnoses and delayed identification [1,8,9]	Adult services benefit from flexible, developmentally informed assessment models that incorporate retrospective self-report and life-course pattern analysis when childhood documentation is unavailable

Genetic contributions and developmental influences

Population-based studies indicate that autism demonstrates substantial heritability, reflecting a strong genetic contribution to neurodevelopmental variation rather than a deterministic model. Twin, adoption, and family studies consistently estimate heritability between approximately 56% and 95%, depending on methodology and phenotypic definitions [39-41]. Within a dimensional framework, these findings support the concept of a broader autism phenotype, in which heritable variation contributes to diversity in social cognition, communication style, and cognitive processing across the general population [10,11].

The proportion of variance not attributable to genetic factors highlights the role of nonshared environmental, epigenetic, and developmental influences [39-41]. These factors appear to shape neurodevelopmental trajectories through dynamic interaction with genetic variation rather than acting as independent determinants [39-41].

The limited predictive value of current polygenic models further underscores the probabilistic and multifactorial nature of autistic traits. Similar genetic profiles may be associated with different developmental trajectories and patterns of expression across individuals [42,43], reinforcing the distinction between genetic liability and fixed outcomes. This variability supports understanding autism as a heterogeneous neurodevelopmental profile rather than a single biological pathway.

From a psychiatric perspective, recognizing autism as emerging from multifactorial developmental influences helps contextualize the heterogeneity observed in adulthood [2,10,44]. Diverse trajectories, shaped by genetic variation and environmental context, may give rise to subtle, internally compensated, or nonstereotypical adult presentations that differ from models historically derived from early childhood descriptions [1,2,13,18], which emphasized more externally observable features.

This variability may contribute to underrecognition in adult psychiatric settings, particularly when clinical reasoning relies primarily on externally visible behavioral indicators. Integrating developmental history with attention to phenotypic diversity supports more nuanced adult assessment and individualized clinical formulation, especially when developmental information is incomplete or reconstructed retrospectively.

Neurobiology

Neurobiological research in autism has identified patterns of variation across multiple levels of brain organization, including structural connectivity, functional network integration, synaptic signaling, and cortical development. Rather than reflecting a single biological pathway, current evidence supports a heterogeneous and distributed neurodevelopmental profile involving large-scale brain networks related to social cognition, executive functioning, and sensory processing [45-47].

Structural neuroimaging studies describe variability in cortical thickness, white matter microstructure, and long-range connectivity, particularly in frontotemporal and frontoparietal circuits [48,49]. Functional magnetic resonance imaging studies have reported differences in intrinsic connectivity within and between networks such as the default mode network, salience network, and other systems associated with social processing [46,47,50]. Findings vary across age groups, cognitive profiles, and methodologies, with limited replication in some areas [46,47,51]. Overall, this suggests that neurobiological organization reflects developmental divergence and network-level variability rather than a uniform structural pattern [51].

At the synaptic and molecular levels, genetic and translational research implicates pathways involved in synaptic development, plasticity, and excitatory-inhibitory balance [52]. Genome-wide association studies support a polygenic architecture, in which multiple common variants contribute probabilistically to autistic traits [43]. These mechanisms interact dynamically with developmental and environmental influences across the lifespan [42,43].

Lifespan research indicates that neurodevelopmental trajectories vary substantially between individuals [15,46]. Neural patterns may change and reorganize, or be functionally compensated across adolescence and adulthood [15,46]. Normative modeling studies further highlight marked interindividual variability in cortical trajectories, supporting a model of neurodevelopmental diversity rather than uniformity [15,51].

Neurobiological findings remain heterogeneous and, in some areas, inconsistent across studies [51]. Variability in sampling, age ranges, co-occurring conditions, and methodological approaches contributes to divergent results, particularly in functional connectivity research [46,47]. In addition, many studies rely on relatively small or cross-sectional samples, limiting conclusions about long-term developmental trajectories. No single neurobiological marker has demonstrated sufficient sensitivity or specificity for identification [50], and current models are best understood as evolving frameworks rather than definitive biomarkers.

From a psychiatric perspective, these findings provide a developmental context for understanding the heterogeneity observed in adulthood. Variations in network integration, sensory processing, and executive regulation may be associated with experiences such as social fatigue, heightened sensory sensitivity, cognitive overload, structured coping strategies, or internalizing presentations rather than overt behavioral markers. These patterns may reduce external visibility and contribute to underrecognition in adult psychiatric settings [2,3,26,27,30].

Neurobiological models should therefore be integrated with developmental, dimensional, and contextual perspectives when formulating adult presentations. In this way, they contribute to a more nuanced understanding of phenotypic diversity across the lifespan rather than serving as reductionist explanations [2].

Presentation in adulthood

Autism in adulthood reflects the persistence of core neurodevelopmental traits expressed within changing contextual demands and adaptive processes, resulting in heterogeneous developmental trajectories [3,14,53]. Differences in social communication, focused and repetitive interests, and sustained cognitive and adaptive effort remain central; however, their visibility and functional impact vary across development [2,19]. This variability reflects the interaction between neurodevelopmental configuration and environmental context, contributing to the complexity of recognition in adult psychiatric settings [3,13,53].

Social Communication and Camouflaging

In adulthood, social differences are often less evident as overt behavioral features and more commonly appear as subtle variations in pragmatic language, implicit social inference, nonverbal communication, and increased cognitive effort during interpersonal interaction [19,21,54]. In clinical settings, these differences may emerge through reports of fatigue, effortful social engagement, or difficulty navigating implicit expectations [19,21,54].

Differences in nonverbal communication have also been described, including variations in eye contact, facial expression, and prosody that may not consistently align with contextual norms, as well as differences in gesture use or communicative emphasis [3,55]. These patterns may influence perceptions of social competence even when verbal content is appropriate [3,55]. Differences extend not only to the expression of social signals but also to their interpretation, with autistic adults often describing increased effort in processing implicit cues, emotional nuance, and rapidly shifting conversational demands [54]. The double empathy framework suggests that these differences may reflect bidirectional mismatches in communicative styles between autistic and nonautistic individuals [56].

A growing body of research describes camouflaging as a set of strategies used to adapt to social expectations [21,29]. This includes masking (the suppression or modification of behaviors), compensatory strategies (cognitive or behavioral adaptations that support social participation), and assimilation (adopting socially expected interaction styles) [21,22,28]. These strategies may involve imitation, modulation of prosody or affect, and reliance on internalized conversational scripts [21,22,28].

Meta-analytic evidence indicates that camouflaging is associated with higher levels of anxiety, depressive symptoms, suicidality, and psychological distress [23,57,58]. While these strategies may facilitate social participation or reduce immediate interpersonal friction, they may also involve sustained cognitive and emotional effort. Over time, this effort may contribute to internal burden, even when external functioning appears socially normative [28,59].

Focused Interests and Repetitive Patterns

Highly focused interests are consistently reported across the lifespan and may remain central to adult functioning and identity [53,60]. In adulthood, these interests may support expertise, vocational engagement, and identity development, particularly when aligned with structured domains or areas that value detailed information processing [60,61].

Preferences for predictability and environmental stability may also persist, reflecting differences in cognitive and behavioral flexibility [27,53]. Unexpected changes in routines or environments may be experienced as disruptive, leading to distress, avoidance, or adaptive reorganization [53]. From a neuropsychological perspective, these patterns have been associated with differences in executive functions, including cognitive flexibility, inhibitory control, planning, and self-regulation [27,62].

When aligned with occupational demands or personal goals, focused interests may enhance adaptive functioning and professional development. In contrast, when environments require sustained flexibility or limit the expression of these interests, individuals may experience interpersonal or occupational challenges [60,62].

Internalizing Symptoms, Compensation, and Executive Function

Autistic adults frequently seek psychiatric care for anxiety, depressive symptoms, and chronic stress-related difficulties [7,30,31,63]. These presentations often reflect the interaction between neurodevelopmental traits and the cumulative demands of adult social and occupational environments [30,31,63].

Differences in executive functioning, particularly in cognitive flexibility, planning, task initiation, and organization, may contribute to challenges in occupational settings or mismatches with external expectations [27,62]. Sensory processing differences, including heightened sensitivity or reduced sensory filtering, may further increase cognitive load and environmental stress [26]. Together, these factors can increase the effort required to manage everyday demands.

Sustained social compensation and camouflaging, as described previously, may contribute to cumulative psychological load over time [22,23,28]. Emerging research has linked prolonged compensatory effort and chronic stress exposure with experiences described as autistic burnout. This construct refers to states of significant exhaustion, reduced capacity to manage daily demands, and increased sensory sensitivity following sustained adaptation to environmental expectations [24,64,65].

Autistic Burnout

Autistic burnout has been described in qualitative and emerging quantitative research as a state of significant exhaustion, reduced capacity to manage daily demands, and increased sensory or emotional sensitivity following prolonged efforts to meet environmental expectations [64,65]. Although it may overlap with depressive symptomatology, it is more commonly characterized by cognitive depletion, shutdown-like responses, and reduced tolerance to sensory input, rather than persistent low mood or anhedonia [64].

In clinical settings, burnout may be reflected in a decline from previously stable levels of functioning, reduced social tolerance, and increased sensory reactivity [64]. While its empirical definition continues to evolve and formal diagnostic criteria are not established, the concept has growing relevance in adult services and may be useful in guiding clinical understanding and assessment [59,65].

From a clinical perspective, recognizing autistic burnout has important implications for immediate intervention [59,64,65]. Clinical evaluation may benefit from considering recent changes in functioning, cumulative adaptive demands, and the role of prolonged camouflaging. Differentiating burnout from primary mood or anxiety disorders may help avoid unnecessary pharmacological escalation, while initial management may include reducing environmental demands, supporting sensory regulation, and providing psychoeducation and validation to facilitate recovery and engagement with care [64,65]. Table 2 summarizes key domains of adult autism presentation and highlights factors that may reduce the visibility of autistic characteristics while contributing to underrecognition in adult psychiatric settings.

**Table 2:** Core domains of adult autism presentation and factors influencing recognition Domains summarize commonly described patterns of adult autism presentation and factors that may influence recognition

Domain of adult presentation	Typical manifestations in adulthood	Factors reducing visibility
Social communication differences	Subtle differences in pragmatic language, conversational reciprocity, interpretation of implicit social cues, and sustained cognitive effort during interpersonal interaction. Nonverbal communication may include variations in eye contact, facial expression synchrony, and prosody [3,44,54]	Social learning and camouflaging strategies may produce superficially typical interaction in structured settings. Brief interactions may capture observable behavior but not the cognitive effort required to sustain social interaction [21,28]
Interpretation of social information	Greater effort interpreting implicit social signals, emotional nuance, or rapidly shifting conversational expectations. Social interaction involve conscious analytical processing rather than automatic inference [54,55]	Differences may be interpreted as shyness, introversion, or social anxiety rather than reflecting neurodevelopmental variation [30]
Focused interests	Highly focused interests involving deep knowledge, pattern recognition, or engagement with specific topics. These interests may support identity development and professional expertise [60,61]	These patterns may be interpreted as a meticulous or perfectionistic working style, which can reduce their recognition as part of a broader neurodevelopmental profile [14]
Cognitive and behavioral inflexibility	Preference for predictability, reliance on routines, and challenges adapting to unexpected changes in environments or expectations. Often associated with differences in executive processes such as cognitive flexibility and planning [27,62]	In adulthood, these patterns may be interpreted as personality style, perfectionism, or coping strategies rather than neurodevelopmental differences [30]
Executive functioning differences	Differences in task initiation, organization, planning, and cognitive flexibility. These may manifest as occupational challenges, alternative ways of managing tasks, or variable productivity despite overall capability [27,62]	Executive differences may be interpreted as mood-related difficulties, stress responses, or motivational factors when developmental history is not explored [30]
Sensory processing differences	Heightened sensory sensitivity, reduced sensory filtering, or increased perceptual load in complex environments. Sensory stimuli such as noise, light, or crowded spaces may increase cognitive load or sensory discomfort [26]	Sensory experiences may be interpreted as anxiety, irritability, or stress reactivity rather than differences in sensory processing [31]
Camouflaging and compensatory strategies	Masking autistic behaviors, imitation of social scripts, modulation of prosody or facial expression, and development of cognitive strategies to navigate interaction demands [21,28,29]	Camouflaging may reduce externally observable markers of autism, while increasing internal cognitive and emotional load [23,58]
Internalizing symptoms	Anxiety, depressive symptoms, chronic stress, and psychological distress are common reasons for psychiatric consultation among autistic adults [7,31,66]	Psychiatric symptoms may dominate clinical presentation, obscuring underlying neurodevelopmental patterns if developmental history is not systematically assessed [1]
Autistic burnout	Significant exhaustion, reduced functional capacity, heightened sensory sensitivity, and decreased tolerance for social demands following prolonged compensatory effort. May involve reductions in previously stable adaptive functioning [64,65]	Burnout may be interpreted as depressive relapse, occupational stress, emotional dysregulation, or general fatigue if the role of sustained camouflaging and environmental mismatch is not recognized [64]

Course across adulthood

Autism in adulthood reflects the persistence of core neurodevelopmental features alongside changes in how these are expressed as social, academic, and occupational demands increase. Early manifestations are often interpreted as shyness, introversion, giftedness, or personality differences, contributing to limited recognition during childhood and adolescence [1,21]. As a result, psychiatric consultation in adulthood frequently occurs in the context of emotional distress, adaptive challenges, or occupational and relational strain, particularly when sustained compensatory strategies become difficult to maintain [30,31,57,67,68].

The course across adulthood is characterized by variability in functioning, shaped by the interaction between cognitive and sensory profiles, environmental demands, and access to support and accommodations [3,69,70]. Longitudinal studies suggest relative stability of core traits alongside context-dependent changes in adaptive functioning [3,69,70]. However, much of the available literature is limited by sampling biases and historical diagnostic frameworks, which may restrict generalizability to current adult populations [44].

Late identification may provide explanatory clarity and facilitate reinterpretation of prior experiences, often reducing self-blame [37]. At the same time, it may highlight the cumulative impact of prolonged adaptation in environments that did not recognize neurodevelopmental differences, a process increasingly described as autistic burnout [64,65].

The transition to adulthood represents a period of increased vulnerability. Expanding expectations for autonomy, social navigation, executive functioning, and self-management often coincide with limited availability of adult-oriented services [13,18,68]. Structural barriers include long waiting times, restrictive eligibility criteria, fragmented services, and limited clinician training in adult autism assessment [13,18]. These factors, along with reduced environmental structure and limited vocational support, are associated with underemployment, prolonged dependence, and increased reliance on crisis-based care. Importantly, these outcomes often reflect systemic constraints rather than individual limitations [18,68,71,72].

During adolescence and early adulthood, individuals are required to navigate implicit social norms, increasing task complexity, and independent living demands. These expectations may require sustained cognitive and adaptive effort, particularly in the context of differences in social cognition, behavioral flexibility, sensory processing, or executive functioning [27]. Anxiety and depressive symptoms are common during this stage and appear to reflect the interaction between chronic adaptive demands and misaligned environments [31,67].

Adult outcomes are highly variable. Long-term studies suggest that a minority of autistic individuals achieve full independent living, with approximately 20%-30% living independently and many others requiring varying levels of support [3,69]. Competitive employment rates remain low, with fewer than one-third obtaining competitive employment in many cohorts, even among those with average or above-average intellectual ability [68,69,71]. When employment is achieved, underemployment and role mismatch are common and are often related to structural barriers, workplace inflexibility, and stigma rather than differences in ability [72].

Although many autistic adults report a strong interest in social connection, differences in communication style and relational reciprocity may contribute to experiences of isolation or interpersonal strain [16]. Quality of life is strongly influenced by environmental factors, with greater social support, lower chronic stress, and inclusive work environments associated with more positive outcomes [16,17].

Research on aging in autism remains limited, particularly beyond midlife. Emerging evidence suggests higher rates of chronic medical conditions, increased cardiometabolic risk, greater frailty, and persistent social isolation in older adults [73-75]. Mortality studies indicate elevated risk, particularly among individuals with co-occurring psychiatric conditions and limited access to sustained support [75].

Overall, adult trajectories are best understood as emerging from the interaction between neurodevelopmental configuration, environmental demands, and access to support. Autism in adulthood is therefore more accurately conceptualized as a lifelong neurodevelopmental profile shaped continuously by context, adaptation, and systemic factors rather than as a fixed or static condition [2,13].

Co-occurring psychiatric and medical conditions

The term co-occurring psychiatric and medical conditions is used to align with established psychiatric terminology while situating these associations within a developmental and interactional framework [7,13,18,30]. Elevated rates of psychiatric and medical conditions in autistic adults have been consistently reported compared with the general population, with approximately 70%-80% experiencing at least one co-occurring psychiatric condition across the lifespan [7], and more than half presenting at least one chronic medical condition [74]. A substantial proportion also experience multiple concurrent conditions, contributing to increased care complexity, higher healthcare utilization, and elevated risk of adverse outcomes, including a two- to threefold increase in mortality risk [7,30,75]. The prevalence and clinical impact of these conditions vary across individuals and are shaped by developmental trajectories, social context, timing of recognition, and access to care [7,13,18].

Neurodevelopmental Co-occurring Conditions

Neurodevelopmental conditions frequently co-occur with autism and reflect partially shared genetic and developmental substrates [7]. Attention-deficit/hyperactivity disorder (ADHD) is a neurodevelopmental condition characterized by inattention and/or hyperactivity-impulsivity, with prevalence in autistic adults ranging from approximately 30%-80% [76-78]. ADHD is typically associated with variable attention and distractibility, whereas attentional patterns in autism more often involve sustained, interest-driven engagement [27,76,78]. When both co-occur (often referred to as AuDHD), overlapping features may complicate recognition, particularly without a longitudinal developmental perspective [7,27].

Learning disability is a neurodevelopmental condition characterized by significant limitations in intellectual functioning and adaptive behavior. It is present in a substantial minority of autistic individuals and is associated with greater support needs and increased clinical complexity [44,79]. Specific learning differences may also persist into adulthood, contributing to academic and occupational challenges, including among individuals with average or above-average intellectual ability [62,80].

Psychiatric Conditions in Adulthood

Depressive and anxiety disorders are among the most common co-occurring conditions, characterized respectively by persistent low mood and anhedonia, and by excessive fear or worry, with lifetime prevalence estimated at approximately 30%-40% [31,66]. These are closely associated with chronic social stress, sensory overload, prolonged compensatory effort, and experiences consistent with minority stress processes [13,16,18,81].

Obsessive-compulsive disorder (OCD) is characterized by intrusive, unwanted thoughts (obsessions) and repetitive behaviors or mental acts (compulsions) performed to reduce distress. Autism-related routines may resemble compulsions but typically serve regulatory or predictability-enhancing functions, whereas OCD compulsions are driven by intrusive, ego-dystonic thoughts [82]. Careful assessment of subjective experience and developmental history is essential.

Bipolar and psychotic disorders are characterized, respectively, by episodic mood disturbance with depression, manic or hypomanic features, and by alterations in reality testing such as delusions or hallucinations. Both have been reported at elevated rates in autistic individuals, although estimates vary [83,84]. Phenomenological overlap may arise from shared features such as social withdrawal, atypical affect, rigidity of thinking, and unusual perceptual experiences, increasing the risk of both over- and underdiagnosis [30,67,83]. Longitudinal assessment is therefore essential to differentiate neurodevelopmental characteristics from episodic psychiatric conditions [67].

Personality Disorders and Dimensional Overlap

Personality disorders are characterized by enduring patterns of inner experience and behavior that deviate from cultural expectations, are pervasive and inflexible, and lead to distress or impairment in functioning [3]. Diagnoses of personality disorder are frequently assigned in autistic adults, particularly obsessive-compulsive personality disorder (OCPD), borderline personality disorder, and schizoid personality [1,30,67]. Borderline personality disorder is characterized by instability in affect, self-image, and interpersonal relationships, schizoid personality disorder by social detachment and restricted emotional expression, and OCPD by a pervasive pattern of preoccupation with order, control, and perfectionism. In autistic individuals, apparent overlap may arise, as emotional intensity and relational instability may resemble borderline features, social withdrawal and restricted affect may resemble schizoid traits, and preferences for order or predictability may resemble OCPD traits, although these are often better understood as adaptive or regulatory strategies rather than personality pathology [3,44,82]. More broadly, overlap across these conditions may reflect shared dimensional traits, including social detachment, emotional regulation differences, cognitive rigidity, and atypical interpersonal patterns, rather than distinct categorical conditions [3,44,82]. In women, overlap with borderline personality disorder has been frequently reported [21,32], which may relate to shared features as well as gendered recognition bias, camouflaging, and misinterpretation of neurodevelopmental differences. Failure to assess developmental history increases the risk of misclassification, underscoring the importance of a developmental formulation before attributing difficulties to fixed personality structures [3,21,32].

Suicidality and the Limits of Traditional Risk Models

Suicidality refers to the presence of suicidal ideation, plans, or attempts, and represents a major clinical concern in autistic adults, with markedly elevated rates compared with the general population. Lifetime suicidal ideation has been reported in approximately 60%-65% of autistic adults, and suicide attempts in around 20%-30% [57,75].

Traditional risk models may not fully capture suicide risk, as they often prioritize acute psychiatric symptoms. In autistic adults, risk may also be associated with chronic social exclusion, cumulative stress, prolonged camouflaging, unmet support needs, and environmental overwhelm [21,45,57]. These findings suggest that risk assessment may benefit from integrating developmental, relational, and systemic factors alongside standard psychiatric evaluation.

Medical and Multisystem Conditions

Autism is also associated with a range of medical and multisystem conditions that contribute to overall health complexity. Epilepsy, defined by recurrent unprovoked seizures, is more prevalent in autistic individuals, particularly among those with higher support needs [85]. Tic disorders, characterized by sudden, rapid, recurrent motor movements or vocalizations, share overlapping neurodevelopmental features with autism, including repetitive behaviors and reduced voluntary control [86].

Sleep disturbances, including insomnia (difficulty initiating or maintaining sleep) and circadian dysregulation (altered sleep-wake timing), are common in autistic individuals [87]. Gastrointestinal conditions and metabolic factors, such as overweight and obesity, are also frequently reported and may be influenced by sensory preferences and lifestyle factors [74,88].

Motor coordination differences in autism may include difficulties with fine- and gross-motor control, balance, and motor planning, affecting daily functioning and participation. Immune-related conditions, including increased rates of allergic and inflammatory disorders, have also been reported, suggesting links between neurodevelopmental processes and immune regulation. Feeding-related challenges may involve sensory sensitivities, restrictive eating patterns, and interoceptive differences, contributing to nutritional and health-related concerns [89-91]. Table 3 summarizes frequently reported co-occurring psychiatric and neurodevelopmental conditions in autistic adults.

**Table 3:** Frequently reported co-occurring conditions in autistic adults Prevalence estimates vary substantially according to sampling methods, diagnostic criteria, and cohort characteristics. Conditions listed are not mutually exclusive and frequently co-occur. Clinical considerations are intended to support developmental formulation while maintaining independent diagnostic evaluation and evidence-based treatment of co-occurring conditions

Clinical domain	Condition	Estimated prevalence	Key clinical features
Neurodevelopmental regulation	Attention-deficit/hyperactivity disorder	~30%-60% (up to 70%-80% in clinical samples)	Frequent co-occurrence with autism, with overlap in executive dysfunction, attentional regulation, and inhibitory control [76-78]
	Learning disability	~20%-40% (lower in recent adult cohorts)	More prevalent in earlier clinical cohorts; lower rates in more recent cohorts reflecting increased recognition of autism without learning disability [3,14,53]
	Specific learning disorders	Common/frequently reported	Difficulties in reading, writing, and mathematics are frequently reported in autistic individuals and are often identified in educational contexts [3,53]
Affective disorders	Major depressive disorder	~30%-40%	Common in autistic adults and associated with chronic social stress, loneliness, and social camouflaging [31,58,66]
	Bipolar disorder	~5%-8%	Diagnostic overlap with behavioral activation, circadian dysregulation, and intense goal-directed behavior [7,84]
Anxiety and compulsivity spectrum	Anxiety disorders	~40%-50%	Includes social anxiety, generalized anxiety disorder, and phobias, with considerable variability depending on age and diagnostic method [7,30,31]
	Obsessive-compulsive disorder	~10%-20%	Requires differentiation from repetitive behaviors intrinsic to autism [7,82]
Psychotic spectrum	Psychotic disorders	~5%-10% (higher in clinical samples)	Associated with increased clinical complexity and functional impairment; possible shared neurodevelopmental vulnerability [7,83]
Personality-related traits	Obsessive-compulsive personality disorder	Elevated prevalence reported (~20%-30%)	Characterized by cognitive rigidity, perfectionism, and high need for control [30]
	Borderline personality disorder	Increased prevalence and diagnostic overlap reported	Phenotypic overlap in emotional dysregulation, interpersonal instability, and social cognition [32]
Eating and behavioral regulation	Anorexia nervosa and related eating disorders	Increased prevalence reported, particularly among autistic women	Shared traits include cognitive rigidity, sensory sensitivities, and restrictive behavioral patterns [92]
Self-harm and suicidality	Suicidal ideation or behavior	Up to ~60%-65% lifetime suicidal ideation (higher in clinical and late-diagnosed samples)	Elevated risk associated with depression, anxiety, and social exclusion in autistic adults [57,63,75]
Sleep and circadian regulation	Sleep disorders	~50%-80%	Insomnia, sleep fragmentation, and circadian rhythm alterations commonly reported in autism [87]

Discussion

Challenges for Psychiatric Practice

Psychiatric practice has historically conceptualized autism primarily within childhood frameworks, with recognition systems, training models, and service structures largely oriented toward early identification. As a result, adult mental health services often lack the conceptual tools and organizational pathways needed to recognize autism in adulthood, particularly in individuals with subtle, internalizing, or camouflaged presentations [1].

Assessment in adulthood presents additional complexities. A central challenge is the reliance on observable behavior during time-limited evaluations [1,21,28]. In autistic adults, outward social competence may coexist with substantial internal cognitive and emotional effort related to sustained compensatory strategies [21,28]. As a result, interviews that prioritize surface-level functioning may underestimate distress, overlook the costs of adaptation, and misattribute difficulties to personality traits or other psychiatric conditions [20-22].

Diagnostic overshadowing is a frequent source of error [3,7,18,30]. Emotional and interpersonal difficulties may be treated as isolated conditions without considering their developmental context. Conversely, once autism is recognized, there may be a tendency to attribute all subsequent difficulties to it, potentially overlooking co-occurring conditions that require independent assessment and treatment [7,18,30]. In both cases, the difficulty lies in reducing complex presentations to a single explanatory model, which may contribute to fragmented care and suboptimal treatment planning [7,18,57].

Adult autism also highlights the limitations of strictly categorical diagnostic models when applied without developmental context [2,10]. Many individuals present with constellations of traits that span mood, anxiety, personality, and neurodevelopmental domains [2,3,10]. When interpreted rigidly, categorical frameworks may fragment this continuity into multiple labels over time, obscuring developmental patterns and lived experience [2,3,10].

Training structures represent an additional challenge. Educational programs have traditionally focused on childhood presentations and observable behavioral markers, with less emphasis on adult phenomenology or later-life recognition pathways [1,3]. As a result, clinicians may have limited exposure to the diverse ways autism presents in adulthood, particularly when the developmental history is incomplete. This gap reflects systemic training patterns rather than individual limitations and highlights the need for continued professional development [4].

Service-level factors further contribute to these challenges. Adult autism-informed services are often limited, referral pathways are fragmented, and waiting times are prolonged. As a result, individuals may access care primarily during periods of acute distress rather than through preventive or continuous support models. This contributes to increased use of emergency and psychiatric services without consistently providing developmentally informed care [13,18].

Finally, psychiatric practice must integrate medical and neurodiversity-informed perspectives. The neurodiversity paradigm conceptualizes autism as a form of natural variation in human neurodevelopment rather than solely as a disorder, emphasizing heterogeneity, strengths, and the role of social and environmental context [13]. At the same time, psychiatry retains responsibility for addressing distress and risk when present. The task is, therefore, integrative: recognizing autism as a neurodevelopmental profile while avoiding reductionist models and ensuring appropriate assessment and treatment of co-occurring conditions [2,13].

These challenges highlight the need for both conceptual and structural adjustments in adult psychiatric practice. Improving recognition requires moving beyond behavior-focused assessment, incorporating developmental formulations, strengthening clinician training, and improving service integration. Without these changes, many autistic adults may continue to experience delayed recognition and fragmented care [1,3,18].

Clinical Implications for Adult Psychiatric Assessment and Formulation

The high prevalence and diversity of co-occurring conditions in autistic adults highlight the limitations of fragmented, categorical diagnostic approaches. When autism is not recognized as the organizing developmental context, clinical care may involve serial rediagnosis, symptom-focused treatment escalation, or polypharmacy without addressing underlying person-environment mismatch [7,18,30].

An integrated formulation requires moving beyond cross-sectional symptom assessment. Many autistic adults present after years of adapting to unrecognized neurodevelopmental differences [1,21,28]. Subtle or compensated presentations may reduce overt behavioral markers while increasing internal distress and cumulative burden [21,22,37]. As a result, observable functioning may not reflect the level of effort required to maintain it [21,28,59].

Dimensional and developmental formulations offer a complementary perspective by situating psychological distress within longitudinal trajectories and functional context [3,10]. Rather than focusing exclusively on categorical diagnoses, assessment may consider patterns of social communication, sensory processing, cognitive flexibility, affective regulation, and adaptive functioning across the lifespan. This approach supports recognition of compensated or subthreshold presentations and clarifies the relationship between neurodevelopmental variation and later psychiatric symptoms. The goal is not to replace existing diagnostic systems but to enhance clinical formulation in a way that is developmentally informed and clinically meaningful [2,7,10].

Within this framework, clinical formulation may benefit from distinguishing three interacting domains: core neurodevelopmental characteristics, co-occurring psychiatric conditions, and environmentally mediated or cumulative stress-related difficulties. Differentiating these domains may reduce fragmented care and support more coherent treatment planning.

Assessment in adult psychiatry may, therefore, benefit from routinely considering autism within diagnostic formulation, particularly in individuals presenting with recurrent depression, anxiety, chronic suicidality, emotional dysregulation, or complex personality presentations [7,57]. This is especially relevant when these presentations coexist with longstanding social differences, focused interests, sensory sensitivities, or a persistent sense of social divergence [1].

A thorough developmental history remains central, even when formal documentation is unavailable. Assessment may include exploration of early social experiences, patterns of interests, academic trajectory, peer relationships, and sensory sensitivities [3,9]. When records are limited, retrospective reconstruction through patient narrative and collateral information, when available, may provide clinically meaningful insight [9]. Self-report is also valuable, particularly when camouflaging has obscured earlier recognition [21].

Assessment should also explore compensatory strategies [21,28]. Many autistic adults describe sustained effort to monitor behavior, interpret social cues, and adapt responses in social contexts [21,28]. Direct inquiry into social fatigue, postinteraction exhaustion, and experiences of “performing” socially may reveal clinically relevant strain [21,23,28]. Periods of reduced functioning or profound exhaustion may also reflect autistic burnout rather than primary mood or anxiety disorders [64,65].

Clinical encounters may benefit from practical adaptations, including reducing sensory load, using clear and explicit communication, structuring interviews, and allowing additional processing time. These adjustments can improve both assessment and therapeutic engagement [18,38].

Screening tools such as the Autism Spectrum Quotient, Social Responsiveness Scale, and Ritvo Autism Asperger Diagnostic Scale-Revised may be useful adjuncts but should not replace comprehensive clinical evaluation. Overreliance on screening may lead to false negatives in individuals who camouflage extensively and false positives in those with overlapping psychiatric features [23,29].

Management of autistic adults often benefits from interdisciplinary collaboration, including psychiatry, psychology, primary care, and allied health services. Integrated care models that address mental health, physical health, and functional needs may support more stable outcomes and reduce reliance on crisis-based care [18,53].

Improving recognition in adulthood also requires adjustments in training and service design. Greater inclusion of adult autism in psychiatric education and accessible diagnostic pathways may reduce delays in recognition and improve care delivery [1,3].

Overall, integrating developmental, dimensional, and contextual perspectives into psychiatric assessment supports more coherent, person-centered, and longitudinally informed care. These considerations highlight the importance of combining clinical and developmental frameworks in understanding adult autism. Future research should further examine longitudinal trajectories, refine assessment approaches in adulthood, and evaluate models of care that address both neurodevelopmental and psychiatric needs.

Limitations

This review has several limitations. First, as a narrative review, it provides a conceptual synthesis of heterogeneous evidence rather than a comprehensive systematic analysis.

Second, much of the available literature is derived from high-income Western countries, which may limit the generalizability of findings to other sociocultural and healthcare contexts.

Third, there is limited representation of gender-diverse and racially marginalized autistic adults in the current literature. This underrepresentation likely reflects structural barriers to recognition and care rather than true differences in prevalence.

Finally, developmental history in adulthood is often incomplete or difficult to reconstruct, particularly in individuals who engaged in camouflaging from an early age. Accordingly, developmental information should be interpreted alongside self-report and contextual accounts when available.

## Conclusions

Autism in adulthood remains underrecognized in psychiatric practice despite strong epidemiological evidence of its prevalence and lifespan persistence, particularly among individuals with subtle, internalizing, or camouflaged presentations. This underrecognition reflects the limitations of historically childhood-focused models and contributes to fragmented care and adverse outcomes. Conceptualizing autism as a heterogeneous and lifelong neurodevelopmental profile, while acknowledging phenomena such as social camouflaging and autistic burnout, supports a more accurate understanding of adult presentations and their clinical implications. Recognizing autism does not replace established psychiatric diagnoses but provides a developmental framework that enhances formulation, risk assessment, and treatment planning while helping to avoid diagnostic overshadowing. Routine consideration of autism in adult psychiatric assessment, alongside attention to adaptive costs and environmental fit, may improve engagement and continuity of care. Overall, integrating developmental and dimensional perspectives may promote more coherent, equitable, and effective psychiatric services for autistic adults.
